# Make Modern Microbiology Matter More in the 2023 European Society of Cardiology Guidelines for the Management of Infective Endocarditis

**DOI:** 10.1093/cid/ciae222

**Published:** 2024-04-24

**Authors:** Karl Oldberg, Magnus Rasmussen

**Affiliations:** Department of Clinical Sciences, Division of Infection Medicine, Lund University, Lund, Sweden; Department of Clinical Microbiology, Infection Control and Prevention, Office for Medial Services, Lund, Sweden; Department of Clinical Sciences, Division of Infection Medicine, Lund University, Lund, Sweden; Division for Infectious Diseases, Skåne University Hospital, Lund, Sweden

**Keywords:** infective endocarditis, guidelines, blood culture, microbiology, blood culture negative endocarditis

## Abstract

The European Society of Cardiology (ESC) 2023 guidelines for the management of infective endocarditis (IE) stress that a multidisciplinary approach is needed to manage patients with IE. In our view, the guidelines do not include the relevant perspectives from modern microbiology. The diagnostic criteria for IE were changed in the ESC 2023 guidelines, and many IE-causing pathogens are either not clearly defined. Moreover, an improved understanding of the relationship between bacterial species and the risk for IE has not been implemented. The guidelines give detailed and, in our view, incorrect instructions about diagnostic testing in blood culture–negative IE without presenting proper evidence. Other important diagnostic aspects, such as the value of repeated blood cultures and incubation time for blood cultures, are not discussed. We believe that a multidisciplinary collaboration that include microbiologists would have improved these guidelines, and we hope for a future harmonization of diagnostic criteria for IE.

## Teaser

The ESC 2023 guidelines for the management of infective endocarditis (IE) need input from microbiologists. Several aspects of microbiological diagnostics and definitions are not correct. Input from relevant areas of expertise is needed to manage IE and to write guidelines.

Both the 2023 Duke–International Society for Cardiovascular Infectious Diseases (ISCVID) criteria for the diagnosis of infective endocarditis (IE) and the 2023 European Society of Cardiology (ESC) guidelines for the management of IE have recently been published [1, 2]. The 2023 ESC guidelines were published after the Duke–ISCVID criteria and could have included the updated diagnostic criteria presented in the Duke–ISCVID criteria. Instead, the ESC guidelines refer to the modified Duke criteria from 2000 [3] with some minor changes. This has resulted in 2 diagnostic criteria for IE, which is unfortunate for researchers in the field and ultimately for patients.

Whereas the 2023 ESC guidelines stress that a multidisciplinary approach is needed to optimally manage patients with IE, the working group is hardly multidisciplinary and there is no microbiologist in the group. Thus, the guidelines, in terms of microbiological diagnostics, fail to elaborate on potentially important aspects of managing patients with IE [4]. We believe that there is a need for more microbiological input in the future when new guidelines are written.

The 2023 ESC guidelines present diagnostic criteria for IE where some changes in the microbiological aspects were introduced. These include introduction of the term “oral streptococci” instead of “viridans streptococci” and “*Streptococcus gallolyticus*” instead of “*Streptococcus bovis.*” Both changes are problematic since some viridans streptococci, such as *Streptococcus anginosus*, are instead “intestinal streptococci,” and *S. gallolyticus* is just one of several species in the bovis group [5]. It is therefore unclear which streptococci should be regarded as typical IE pathogens according to the 2023 ESC diagnostic criteria. Table 1 summarizes some of the problematic features of the ESC 2023 guidelines together with a short proposal for possible actions that can be taken to address these concerns.

**Table 1.** Problems With Microbiological Aspects of the European Society of Cardiology 2023 Guidelines and Proposed Action

Feature	European Society of Cardiology 2023 Problem	Suggested Alternative
Typical pathogens	“Oral streptococci”	Viridans streptococci
	“*Streptococcus gallolyticus*”	*Streptococcus bovis* group
	No mention of other *Streptococcus*-like pathogens	*Aerococcus*, *Abiotrophia*, *Granilucatella*, *Gemella*, *Corynebacterium jeikeium*, *Corynebacterium striatum*, and *Staphylococcus lugdunensis* as typical pathogens
Blood cultures	“With first and last samples drawn ≥1 h apart”	Makes culturing more difficult without evidence of improved sensitivity; delete this claim
	“Blood cultures should be obtained at 30-minute intervals”	Makes culturing more difficult without evidence of improved sensitivity; delete this claim
	No recommendation on follow-up blood culture	Recommend follow-up blood cultures for *Staphylococcus aureus* to assess likelihood of IE
	No mention of TTP	Mention that a short TTP is linked to higher risk for IE
	No mention of incubation time	Prolonged incubation (>5 d) may be needed for *Cutibacterium* in suspected prosthesis valve IE
Strategy	“Blood cultures remain negative at 48 h”	This is also common in culture-positive IE; wait for 5 days before taking action
	“Systematic serological testing”	Not necessary if antibiotics have been given before blood cultures
	Serology for *Aspergillus*	Not to be used; consider beta-D-glucan or galactomannan
	Serology for *Legionella* and *Mycoplasma*	Extremely rare causes of IE; do not recommend
	“Nonbacterial endocarditis should systematically be considered and assays for” “should be performed”	The diagnostic benefit of these tests is doubtful, and the word “should” is far too strong
Other	Figure 4	A new figure is needed
	Table 9	Revise according to the suggestions presented here

Abbreviations: IE, infective endocarditis; TTP, time to positivity.

The ESC 2023 diagnostic criteria for IE do not make use of the improvements in species determination of bacteria, in particular, streptococci, that have occurred since the first publication of the Duke criteria in 1994. For example, it has become evident that patients with bacteremia with streptococci of the mutans, bovis, or sanguinis groups often have IE, whereas patients with bacteremia caused by anginosus group streptococci rarely have IE [6, 7]. In addition, several pathogens are not mentioned in the ESC 2023 diagnostic criteria despite having a propensity similar to that of *Staphylococcus aureus* or “oral streptococci” to cause IE. Such bacterial genera and species include *Abiotrophia*, *Aerococcus*, *Corynebacterium striatum*, *Corynebacterium jeikeium*, *Gemella*, *Granulicatella*, and *Staphylococcus lugdunensis*, and these should all be regarded as typical pathogens [8]. Some of these pathogens are recognized as typical IE pathogens in the Duke–ISCVID criteria [2].

An entire section (5.3.1) of the ESC guidelines describes standard blood culture practices, which is of limited interest to the clinician and of no use for laboratory personnel. In particular, the guidelines recommend drawing blood cultures at a 30-minute interval, referring to Lamy et al [9]. This reference states that “a 30–60 min interval between samples has been arbitrarily recommended” and that “Li et al (1994) found no difference in blood culture yield whether samples were collected within a 24-h period, either simultaneously or serially” [9], which does not support the recommendation of continued use of the arbitrary sampling interval. It is very impractical to wait between blood cultures, and such a recommendation necessitates firm evidence, which is lacking. What is not mentioned but would have been useful for the reader of the guidelines is that a short time to blood culture positivity in *S. aureus* and *Enterococcus faecalis* bacteremia is associated with increased risk of IE [10, 11]; positive blood cultures drawn at 48–72 hours after the initiation of antibiotic treatment is associated with increased likelihood of IE [12]; and *Cutibacterium acnes*, a not uncommon cause of prosthesis valve IE, grows slowly; therefore, incubation is longer than the standard 120 hours, increasing the likelihood [13] of finding this pathogen.

We have strong doubts regarding the recommendations for laboratory tests in blood culture–negative IE. The guidelines state that when there is no growth in blood cultures within 48 hours in the setting of suspected IE, “depending on local epidemiology, systematic serological testing for *Coxiella burnetii*, *Bartonella* spp., *Aspergillus* spp., *Mycoplasma pneumoniae*, *Brucella* spp., and *Legionella pneumophila* should be proposed.” We believe that such tests can wait since several much more common causes of IE, such as HACEK (*Haemophilus*, *Aggregatibacter*, *Cardiobacterium*, *Eikenella*, and *Kingella*) bacteria, and *C. acnes* will typically take more than 48 hours for blood culture positivity [14, 15]. Moreover, because previous antibiotic treatment is by far the most common cause of blood culture negativity in IE, extensive serological testing is unnecessary in most cases [16]. Some of the agents that the guidelines propose to look for using serology are potential causes of IE, but we find little support for screening for the exceedingly rare IE pathogens *L. pneumophila* and *M. pneumoniae.* In fact, the study by Raoult et al [17], which is referred to in the guidelines, found only 2 cases of *Legionella* IE during 10 years of prospective testing of 1998 patients, and the criteria for establishing the diagnosis of *Legionella* using serology were not described. No case of *M. pneumoniae* IE was found [17]. The recommended “serological testing for *Aspergillus* spp.” remains unexplained in the guidelines, but the referred article [17] only mentions anti-*Aspergillus* immunoglobulin G. To our knowledge, this test has no place in the diagnosis of an acute invasive infection caused by *Aspergillus* spp., instead the *Aspergillus* galactomannan and beta-D-glucan tests that could be of interest in this setting [18] are not mentioned.

Figure 4 in the ESC 2023 guidelines is supposed to explain a microbiological diagnostic algorithm. Several aspects of this figure are very hard to understand, such as how a positive blood culture from which there was no subsequent growth on plates could lead to serology and why antimicrobial susceptibility testing should be performed on DNA amplified by polymerase chain reaction. This figure needs substantial revision.

The ESC 2023 guidelines for the management of IE have several excellent sections. However, a multidisciplinary approach is needed to write complete guidelines and to treat patients with IE. Inclusion of microbiologists and modern microbiology will make a difference.
